# Loneliness in the general population: prevalence, determinants and relations to mental health

**DOI:** 10.1186/s12888-017-1262-x

**Published:** 2017-03-20

**Authors:** Manfred E. Beutel, Eva M. Klein, Elmar Brähler, Iris Reiner, Claus Jünger, Matthias Michal, Jörg Wiltink, Philipp S. Wild, Thomas Münzel, Karl J. Lackner, Ana N. Tibubos

**Affiliations:** 1 Department of Psychosomatic Medicine and Psychotherapy, University Medical Center of the Johannes Gutenberg University Mainz, Zahlbacher Str. 8, D-55131 Mainz, Germany; 2 Center for Cardiology, Cardiology I, University Medical Center of the Johannes Gutenberg University, Mainz, Germany; 3 Preventive Cardiology and Preventive Medicine, Center for Cardiology, University Medical Center of the Johannes Gutenberg University, Mainz, Germany; 4 Center for Thrombosis and Hemostasis, University Medical Center of the Johannes Gutenberg University, Mainz, Germany; 5 German Center for Cardiovascular Research (DZHK), partner site Rhine Main, Mainz, Germany; 6 Institute for Clinical Chemistry and Laboratory Medicine, University Medical Center of the Johannes Gutenberg University, Mainz, Germany

**Keywords:** Loneliness, Depression, Anxiety, Suicidal ideation, Partnership, Prevalence

## Abstract

**Background:**

While loneliness has been regarded as a risk to mental and physical health, there is a lack of current community data covering a broad age range. This study used a large and representative German adult sample to investigate loneliness.

**Methods:**

Baseline data of the Gutenberg Health Study (GHS) collected between April 2007 and April 2012 (*N* = 15,010; 35–74 years), were analyzed. Recruitment for the community-based, prospective, observational cohort study was performed in equal strata for gender, residence and age decades. Measures were provided by self-report and interview. Loneliness was used as a predictor for distress (depression, generalized anxiety, and suicidal ideation) in logistic regression analyses adjusting for sociodemographic variables and mental distress.

**Results:**

A total of 10.5% of participants reported some degree of loneliness (4.9% slight, 3.9% moderate and 1.7% severely distressed by loneliness). Loneliness declined across age groups. Loneliness was stronger in women, in participants without a partner, and in those living alone and without children. Controlling for demographic variables and other sources of distress loneliness was associated with depression (OR = 1.91), generalized anxiety (OR = 1.21) and suicidal ideation (OR = 1.35). Lonely participants also smoked more and visited physicians more frequently.

**Conclusions:**

The findings support the view that loneliness poses a significant health problem for a sizeable part of the population with increased risks in terms of distress (depression, anxiety), suicidal ideation, health behavior and health care utilization.

## Background

Social relationships and social integration are crucial for emotional fulfilment and development over the life span [1]. As social beings, humans have a basic “need to belong" e.g., [2]. Social isolation refers to a lack of social contact, which can be objectively quantified (e.g. living alone, without a partnership). Loneliness, however, is usually defined subjectively as a painfully experienced absence of social contact, belongingness, or a sense of isolation [3]. It refers to a perceived discrepancy between social needs and their availability in the environment [4]. Moreover, loneliness is an emotional state, reflecting the subjective experience of suffering from social isolation [5].

Research on loneliness over the life span concluded that loneliness was highest in late adolescence, gradually decreasing during middle adulthood, and then increasing for late adulthood [6, 7]. In the German Ageing Survey, men and women from 40 to 85 years participated in three waves from 1996 to 2008 in order to find out whether the extent of loneliness of adults in the second half of their lives has changed in Germany. The prevalence of lonely subjects was estimated at 3 to 7% and declined gradually with age, particularly in the oldest group (70-85 years;[8]). With regard to sociodemographic variables, loneliness has been linked to indicators of social integration such as romantic relationships, perceived social support and acceptance [9]. Unmarried individuals indicated feeling lonelier than married individuals [4]. Childlessness had no effect on loneliness in older adults [4]. Yet, living alone was not per se associated with loneliness, instead a smaller social network was an indicator of loneliness, both in men and women [10]. However, the significance of gender in experiencing loneliness is contested: In a study conducted by Tesch-Römer et al. [8] men reported more loneliness than women (from middle to late adulthood). In the KORA Age study which is based on a random sub-sample of a German community sample of over 4000 adults in the age range of 64-94 years from southern Germany, Zebhauser et al. [10] found that the mean level of loneliness did not differ between men and women; only in the oldest participants (>85 years) loneliness was higher in women. As a result, findings are somewhat difficult to integrate due to the heterogeneity of measures ranging from single items inquiring “if a person feels lonely…” to broadly used scales such as the original UCLA loneliness scale with 20 items [11] or the Loneliness scale by de Jong Gierveld and Van Tilburg [12] with 11 items.

Loneliness has been associated with many negative mental health outcomes [13, 14] such as depression, suicidality, reduced positive emotions, poor sleep quality and general health, as well as physiological changes (e.g., increased cortisol awakening response and pro-inflammatory gene expression). Additionally, loneliness was associated with depression, low life satisfaction, and low resilience - particularly in men [10]. It has therefore been considered a major source of psychological stress [15], especially when combined with depression [16]. Especially, the distinction of loneliness from depressive symptoms has been focused by Cacioppo and colleagues [17, 18]. Accordingly, the relation between loneliness and depressive symptoms are reciprocal, emphasizing that both constructs are intimately related but distinct.

Loneliness also aggravates the morbidity and mortality of cardiovascular, cerebrovascular, and other chronic diseases [15]. It has also been related to cognitive decline and Alzheimer’s disease in aging. In a recent meta-analysis, lack of functional and structural social integration predicted a 50% increase in mortality, which is comparable to traditional risk factors [19]. It has remained an issue of debate whether loneliness independently predicts mortality risk after adjusting for health status, health behavior, depression, and social isolation [20]. A recent meta-analysis of longitudinal data on 35,925 participants by Valtorta et al. [21] found a 29% increase for incident coronary heart disease and 32% for stroke in those with poor social relationships. This effect was in the range of other recognized psychosocial risk factors such as job strain. In the English Longitudinal Study of Ageing (ELSA), Shankar et al. [22] found that loneliness was associated with physical inactivity, smoking, and multiple health risk behaviors. In the Health and Retirement Study [23], the authors found that loneliness was significantly and positively associated with physician visits among persons aged 60 years and older. Three major pathways have been discussed by which loneliness affects health [21]: (1) Health risk behaviors such as smoking and physical inactivity, (2) defective immune function and increase of blood pressure, (3) psychological variables such as reduced self-esteem and decreased coping.

Notably, as most studies have used US samples and focused on late adulthood investigating health-related associations of loneliness [8, 10, 23], current prevalence data are limited for research on the link of loneliness to mental health beyond late adulthood for European populations, in particular in Germany. The purpose of this study was to determine prevalence and determinants of loneliness in men and women of the general population from 35 to 74 years and to identify associations with mental health, health behavior and health care utilization. Therefore, the purposes of the present study were (1) to assess the prevalence and determinants of different degrees of loneliness in men and women, and to determine its associations to (2) mental health, (3) health behavior and (4) health care utilization in a large German community sample across the age range from 35 to 74 years.

## Methods

### Study sample

This study investigated the cross-sectional baseline data of the Gutenberg Health Study (GHS) from April 2007 to April 2012 (*N* = 15,010). As specified by Wild et al. [24], the GHS is a population-based, prospective, observational single-center cohort study in the Rhein-Main-Region in western Mid-Germany. The sample was drawn at random from the local registries of the city of Mainz and the adjacent district of Mainz-Bingen. Recruitment was performed stratified in equal strata for gender, residence and for age decades. Eligible participants were aged 35 to 74 years and gave their written informed consent. We excluded 5.8% of the sample due to insufficient knowledge of German language, physical or mental inability to participate. The response rate amounted to 60.3%. It was defined as the recruitment efficacy proportion, i.e. the number of persons with participation in or appointment for the baseline examination divided by the sum of number of persons with participation in or appointment for the baseline examination plus those with refusal and those who were not contactable [28]. Mean age was 54.9 (±11.1); 49.4% were female.

#### Materials and assessment

Cardiovascular risk factors and clinical variables, venous blood samples, blood pressure, and anthropometric measurements were ascertained in the course of the 5-h baseline-examination in the study center by certified medical technical assistants according to standard operating procedures.

#### Measures and questionnaires

All measures used in our analyses are displayed in Table 1, categorized in sociodemographic variables, health related variables, measures of distress, and loneliness.

**Table 1:** Different degrees of subjective loneliness in a German representative sample: Sociodemographic characteristics, health related variables, and distress

	No	Slight	Moderate	Severe	*p*-value*
	loneliness	loneliness	loneliness	loneliness	
N (%)	13,124 (89.5)	720 (4.9)	569 (3.9)	248 (1.7)	
Sociodemographic variables					
Age (years)	55.1 ± 11.1	53.4 ± 10.6	53.0 ± 11.0	53.1 ± 11.1	< 0.0001
Women	48.3	54.9	64.1	66.5	< 0.0001
Partnership	84.7	55.4	54.3	42.1	< 0.0001
Children	85.6	76.3	81.1	77.3	< 0.0001
Living alone	11.7	36.7	36.1	48.5	< 0.0001
Socioeconomic status	13.1 ± 4.4	12.4 ± 4.4	12.0 ± 4.4	11.4 ± 4.0	< 0.0001
Unemployment	38.7	36.1	40.5	45.5	n.s.
Health related variables					
Smoker	18.5	26.1	27.4	31.8	< 0.0001
BMI (kg/m^2^)	27.3 ± 4.9	27.6 ± 5.6	27.1 ± 5.4	28.4 ± 6.5	n.s.
Alcohol gram/day	11.4 ± 16.7	10.3 ± 18.4	9.9 ± 17.8	8.5 ± 19.6	< 0.0001
Antidepressant	4.5	11.0	15.0	24.8	< 0.0001
Anxiolytic	0.8	2.3	1.6	3.3	< 0.0001
Visited physician past month	41.9	48.3	52.3	63.4	< 0.0001
Inpatient treatment past year	13.0	14.7	19.5	21.1	< 0.0001
Distress					
Current depression (PHQ-8 ≥ 10)	5.2	19.3	30.5	52.6	< 0.0001
Generalized anxiety (GAD) > = 3	4.8	12.9	25.7	40.2	< 0.0001
Panic attack (past 4 weeks)	4.6	10.2	13.6	30.0	< 0.0001
Suicidal ideation	5.6	18.9	26.4	41.8	< 0.0001
Type D	20.5	46.5	54.7	55.1	<0.0001

Note: *chi^2^ or Kruskal Wallis test; numerical values with standard deviation are mean scores, values without standard deviation are percentages

Loneliness was assessed by a single item “I am frequently alone /have few contacts” rated as 0 = no, does not apply, 1 = yes it applies, but I do not suffer from it, 2 = yes, it applies, and I suffer slightly, 3 = yes, it applies, and I suffer moderately, 4 = yes, it applies, and I suffer strongly. Loneliness was recoded combining 0 and 1 = no loneliness or distress; 2 = slight, 3 = moderate, and 4 = severe loneliness.

Mental health measures comprised depression, generalized anxiety, panic, suicidality, depersonalization and Type D personality (also known as Distressed Personality).

The Patient Health Questionnaire (PHQ-8; 25), quantifies depression by the frequency of being bothered by each of the 9 diagnostic criteria of major depression over the past 2 weeks, adding up to a sum score between 0 and 27 points. According to [25], caseness was defined by a PHQ-8 sum score of ≥10, achieving a sensitivity of 88% and a specificity of 88% for major depression.

Generalized anxiety was assessed with the two screening items of the short form of the GAD-7 (Generalized Anxiety Disorder [GAD]-7 Scale; [26]). On the two screening items of the GAD-7, subjects rated “Feeling nervous, anxious or on edge” and “Not being able to stop or control worrying”. According to [27], the sum score of the answers (0 = not at all, 1 = several days, 2 = over half the days, and 3 = nearly every day) assesses generalized anxiety with good sensitivity (86%) and specificity (83%).

Following the study by Michal et al. [27], we measured suicidal ideation by the item “In the last 2 weeks, have you had thoughts that you would be better off dead or of hurting yourself in some way?” of the PHQ-9 (PHQ-9; [25, 28]). Significant suicidal ideation was defined when suicidal ideation was present for several days over the past two weeks or more (0 = not at all to 3 = nearly every day).

Panic was assessed by a single item “Did you have a panic attack in the last 4 weeks”. Response mode was dichotomous: 0 = “no, does not apply”, 1 = “yes it applies” [29].

The German version of the Type-D or Distressed Personality scale (ds14; [30] )assesses a pattern consisting of significant negative affectivity (≥10) in conjunction with significant social inhibition (≥10) with 7 items each.

Health behavior included smoking, which was dichotomized into non-smokers (never smoker and ex-smoker) and smokers (occasional smoker, i.e. cigarette/day, and smoker, i.e. cigarette/day).

Health care utilization was also assessed according to the number and kind of physicians visited in the past month and the number of inpatient treatments in the past year. We further inquired the intake of antidepressants and anxiolytics during the past month. Health care utilization variables were recoded (yes/no).

#### Computer-assisted personal interview

During the computer-assisted personal interview (cf. [24]) participants were asked about their alcohol consumption. As defined by Lampert & Kroll, socioeconomic status (SES) ranges from 3 (lowest) to 27 (highest SES). Additionally, the question “Do you live with your partner in a household together?” (no/yes) was administered.

#### Statistical analysis

We reported absolute numbers, percentages, or means with standard deviations. Comparisons between groups (no, slight, moderate and severe loneliness) were done with Kruskal Wallis test or Chi^2^ tests.

In order to investigate the association between loneliness and depression (PHQ-8 ≥ 10), generalized anxiety (GAD-2 ≥ 3) and suicidal ideation, loneliness was used as a predictor in multiple generalized linear models with a binominal distribution and a log link function adjusted for sociodemographic variables. *P*-values are based on 2-tailed tests. No adjustments for multiple testing were performed, as this was an exploratory study. Due to the large number of tests, we recommend to interpret *p*-values with caution taking effect estimates into account. Statistical analyses were performed using SAS for Windows 9.4 TS Level 1 M1 (SAS Institute Inc.) Cary, NC, USA.

## Results

Table 1 compares participants with different degrees of loneliness based on sociodemographic features, health related variables, and distress. A total of 10.5% reported some degree of loneliness: 4.9% were slightly, 3.9% moderately and 1.7% severely distressed by feeling lonely. Overall, mean age of lonely participants was lower. Loneliness was more frequent in women, in participants without a partner or without children. Almost four times as many lonely participants lived alone compared to those without loneliness. SES declined with increasing loneliness. No significant increases were found for previous unemployment, although there was a descriptive trend of a positive link between feeling lonely and previous unemployment.

Concerning health behavior, the proportion of current smokers almost doubled with increasing loneliness. There was no association with BMI. The average alcohol consumption was negatively linked to loneliness. Regarding health care utilization, there were strong increases in the intake of anxiolytics and antidepressants among lonely participants. The majority of participants (63%) with a strong degree of loneliness had visited a physician (vs. 42% with no feelings of loneliness), and 21% (vs. 13%) had had inpatient treatments. Regarding mental health, more than half of the loneliest participants were also depressed (vs. 5% in the group with feelings of loneliness), and over 40% in this group reported anxiety and suicidal ideation (which increased from 6% to 42%) and 30% panic attacks in the past 4 weeks. Type D increased considerably with the degree of loneliness.

**Fig. 1:**
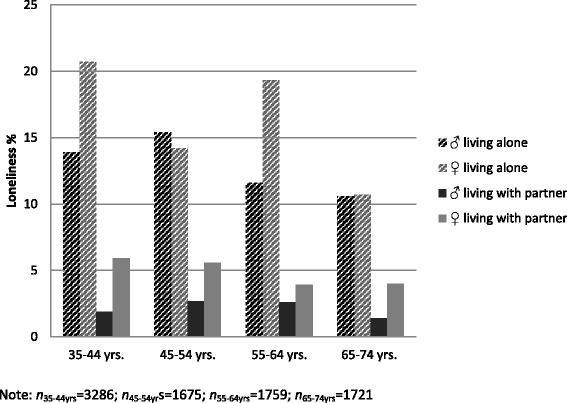
Loneliness across the age range in a German representative sample: men and women living alone or living with partner

Figure 1 presents the proportions of (at least slightly) lonely participants across the age range, separately for men and for women living with or without a partner at the time the participants were interviewed.

Among those living with a partner, loneliness was much lower; few men (1.4 to 2.7%) reported feeling lonely, whereas more than twice as many women reported feeling lonely (up to 5.9%). In contrast, about 10-20% of the participants living alone indicated feeling lonely. Women living alone in the age range 35-44 years (20.7% vs. 13.9%) and 55-64 years (19.3% vs. 11.6%) were considerably more affected by loneliness than men. In the age range of 45-54 years (14.2% vs. 15.4%) and 65-74 years (10.7% vs. 10.6%), no significant gender differences were observed when participant lived alone. Among those living alone, the highest proportion of loneliness was found in the youngest group of women in the range from 35 to 44 years (20.7%), whereas men tended to report heightened loneliness in the range from 45 to 54 years (15.4%).

Table 2 shows significant predictors of distress - separately for depression, anxiety, and suicidal ideation. In addition to loneliness, age, gender, partnership, socioeconomic status, generalized anxiety, panic attacks, and depression were included as predictors in each regression model. Loneliness as one of the predictors of the first regression model almost doubled the likelihood of depression (OR = 1.91). As we expected, generalized anxiety and panic attacks were highly associated with depression, too. Additional factors contributing to a strong overall prediction (c = .90) were lower age and SES. Similarly, loneliness also positively predicted anxiety (OR = 1.21), in addition to lower age, SES, female gender, increased panic attacks, and depression. Prediction of suicidal ideation by loneliness was also substantial (OR = 1.35) in addition to higher age, lower SES, generalized anxiety, panic and depression (overall model, c = .87).

**Table 2:** Prediction of depression, suicidal ideation, and anxiety in a German representative sample by loneliness controlling for demographic variables and other sources of distress (*N* = 15,010)

	Depression (PHQ-8)		Anxiety (GAD-2)				Suicidal ideation		
Variables	OR	95% CI	*p*-value	OR	95% CI	*p*-value	OR	95% CI	*p*-value
Loneliness	1.91	1.74-2.09	< 0.0001	1.21	1.09-1.34	0.0002	1.31	1.19-1.44	<0.0001
Age	0.99	0.98-0.99	0.0004	0.98	0.98-0.99	0.0002	1.03	1.02-1.03	<0.0001
Women	0.94	0.80-1.10	0.4209	1.25	1.06-1.49	0.01	0.92	0.79-1.08	0.3127
Partnership	0.92	0.76-1.11	0.3731	1.02	0.83-1.25	0.8771	0.80	0.67-0.95	0.0117
Socioeconomic status	0.94	0.92-0.96	< 0.0001	0.97	0.97-0.98	0.0043	0.96	0.95-0.98	0.0001
Generalized anxiety	2.91	2.73-3.11	< 0.0001	-			1.30	1.21-1.39	<.0001
Panic attack	2.98	2.42-3.67	< 0.0001	2.69	2.17-3.34	< 0.0001	1.49	1.19-1.86	0.0005
depression	-			1.46	1.42-1.49	< 0.0001	1.29	1.26-1.32	< 0.0001
c-statistic	0.90		0.91				0.87		

Note: *OR* Odds ratio, 95% CI = 95% confidence interval

## Discussion

In a large community sample covering middle to late adulthood (35 to 74 years), we found that one in 10 (10.5%) participants reported some degree of loneliness. As in previous studies e.g. ([7, 8]), loneliness declined with age. Loneliness was more frequent in women, in participants without a partnership, without children and in those living alone. Socioeconomic status declined with increasing loneliness. Living alone was an important determinant of loneliness in men and women. However, the relationship between loneliness, gender and living alone appeared to be complex. Similar to previous findings [10] different patterns of loneliness were observed with regard to age and gender. On the one hand, younger women (below 45 years of age) living without a partner were more affected by loneliness than men. On the other hand, middle-aged men (between 45 and 64 years) were slightly more affected than women. Living without a partner had a stronger impact on younger women and middle-aged men. Finally, in the oldest age group from 65 to 74 years, loneliness declined and no longer differed between men and women living alone. Interestingly, when living with someone, loneliness was comparatively low. However, about twice as many women in partnerships (up to 5.9%) reported feeling lonely in comparison to men (1.4% to 2.7%). Additionally, our data show that living with a partner is linked to loneliness in almost all men and to a slightly lesser extent in women. It might alternatively reflect a reporting bias such as men are less likely to admit to being lonely [31].

Feeling lonely was associated with distress: More than half of the loneliest participants were also depressed (vs. 5% in the group without loneliness). Generalized anxiety, panic attacks and suicidality were strongly associated with loneliness, suicidal ideation increased dramatically from 6% to 42%. There was also a large increase of Type D behavior pattern. This may be due to the previously described relationship deficits in lonely persons and their tendency to experience negative affect [14]. Loneliness remained a clear predictor for depression [17], anxiety and suicidal ideation when we took into account demographic variables and other types of mental distress; odds ratios for depression were 1.9; for suicidal ideation and generalized anxiety, 1.35 and 1.21, respectively. I.e., there was still a 31% increase in suicidality when taking the major demographic and mental health predictors (depression and anxiety) into account. The association between loneliness and suicidality supports the theory [32] that thwarted belongingness and perceived burdensomeness are major determinants of suicidality (see also [33]).

Regarding health care behavior, the majority of participants (63%) with a strong degree of loneliness had visited a physician (vs. 42% without loneliness), and 21% had had inpatient treatments.

Overall, our findings support the view that loneliness poses significant health risks in terms of reduced mental health (depression, anxiety) and increased suicidal ideation [10, 21]. Loneliness also contributed to smoking as an indicator for an unhealthy lifestyle, but not to other risk factors such as alcohol use or diet (BMI). More physician visits, inpatient treatments, and intake of psychotropic medication may be due to subjects` reduced mental health. Taken together, these findings support the view that loneliness should be regarded and inquired about as a relevant health [14] variable on its own.

Strengths of this study reside in the large sample size and the use of standardized self-report instruments to measure distress. Interpretation of our findings is limited by the cross-sectional character of our study which does not permit causal conclusions. Loneliness may contribute to distress and suicidality, however, depressive and anxiety disorders may lead individuals to refrain from social contact out of inhibition and fear. This question of causality has been addressed for depression in a longitudinal study [17] indicating a reciprocal relationship. No similar studies are available for anxiety disorders, yet. As we did not directly assess the size and quality of social networks, we cannot estimate the effect of social isolation in our study which is a confounding variable when investigating loneliness. In line with previous findings [34, 35] our data suggests that younger age groups are more affected by loneliness. Thus, the investigation of the prevalence of loneliness and its impact on mental health in individuals under 35 years old would be desirable. While our findings may be taken as indicative for the validity of our measure of loneliness, we concede that we only used a single item to measure loneliness. The results of our study, which are overall in line with findings in research on loneliness using comprehensive questionnaires, indicate a first step of the external validity of a 1-item-measure of loneliness as reasonable alternative to loneliness scales, especially for large-scale surveys. Future studies will have to prove other aspects of validity of this measure by directly comparing it with other loneliness scales.

## Conclusions

Our findings support the view that loneliness poses a significant health problem for a sizeable part of the population with increased risks in terms of distress (depression, anxiety), suicidal ideation, health behavior and health care utilization.
